# The Role of Glyoxalase-I (Glo-I), Advanced Glycation Endproducts (AGEs), and Their Receptor (RAGE) in Chronic Liver Disease and Hepatocellular Carcinoma (HCC)

**DOI:** 10.3390/ijms18112466

**Published:** 2017-11-20

**Authors:** Marcus Hollenbach

**Affiliations:** Department of Medicine, Neurology and Dermatology, Division of Gastroenterology and Rheumatology, University of Leipzig, Liebigstrasse 20, D-04103 Leipzig, Germany; marcus.hollenbach@web.de; Tel.: +49-341-97-122-00; Fax: +49-341-97-122-09

**Keywords:** ethyl pyruvate, cirrhosis, fibrosis, methylglyoxal, AGEs, CCl_4_

## Abstract

Glyoxalase-I (Glo-I) and glyoxalase-II (Glo-II) comprise the glyoxalase system and are responsible for the detoxification of methylglyoxal (MGO). MGO is formed non-enzymatically as a by-product, mainly in glycolysis, and leads to the formation of advanced glycation endproducts (AGEs). AGEs bind to their receptor, RAGE, and activate intracellular transcription factors, resulting in the production of pro-inflammatory cytokines, oxidative stress, and inflammation. This review will focus on the implication of the Glo-I/AGE/RAGE system in liver injury and hepatocellular carcinoma (HCC). AGEs and RAGE are upregulated in liver fibrosis, and the silencing of RAGE reduced collagen deposition and the tumor growth of HCC. Nevertheless, data relating to Glo-I in fibrosis and cirrhosis are preliminary. Glo-I expression was found to be reduced in early and advanced cirrhosis with a subsequent increase of MGO-levels. On the other hand, pharmacological modulation of Glo-I resulted in the reduced activation of hepatic stellate cells and therefore reduced fibrosis in the CCl_4_-model of cirrhosis. Thus, current research highlighted the Glo-I/AGE/RAGE system as an interesting therapeutic target in chronic liver diseases. These findings need further elucidation in preclinical and clinical studies.

## 1. Pathogenesis of Chronic Liver Disease and Cirrhosis

Chronic liver disease is mainly caused by hepatitis B and C virus infections, alcoholism, nonalcoholic steatohepatitis, or rare autoimmune and hereditary disorders. Thus, liver cirrhosis belongs to the global burden of disease responsible for more than one million deaths p.a. (approximately 2.0% of all deaths) and 31,027,000 disability adjusted life years (DALYs) [1].

The underlying molecular mechanisms for the development of fibrosis, cirrhosis, and portal hypertension have been investigated intensively over the last few decades. Cirrhosis constitutes the end stage of chronic inflammatory liver diseases, resulting in altered liver anatomy (regenerative nodules, hepatocyte ballooning, and fibrosis) [2], reduced liver function, and the elevation of intrahepatic resistance. In addition to the structural alterations, cirrhosis is characterized by an imbalance in vasoactive mediators, indicated by an increase in vasoconstrictors (mainly Endothelin-1) and a decrease in vasodilators (mainly nitric oxide), leading to endothelial dysfunction. As a result of the above-mentioned alterations in cirrhosis, portal hypertension occurs, with the added risk for the development of ascites and esophageal varices [3,4,5].

Since the liver is made up of parenchymal cells (mainly hepatocytes (HEP)) and nonparenchmyal cells (Kupffer cells (KC), hepatic stellate cells (HSC), and liver sinusoidal endothelial cells (LSEC)), all cell types are involved in the development of fibrosis and cirrhosis. Nevertheless, HSC are the main drivers of fibrosis and increased intrahepatic vascular resistance. In this regard, deleterious agents (e.g., alcohol, endotoxins (LPS)) exert toxic effects on the hepatocytes and trigger the production of reactive oxygen species (ROS). ROS are reactive compounds containing oxygen. Besides exogenous ROS (produced through radiation, drugs, smoke, and others), endogenous ROS are formed via multiple mechanisms. Therefore, NADPH oxidase complexes represent the main source of intracellular ROS production [6]. Important ROS comprise, amongst others, hydrogen peroxide, superoxide, and hydroxyl radicals, as well as singlet oxygen. ROS levels can dramatically increase upon stimulation and result in oxidative stress with cell damage, inflammation, apoptosis, and gene alterations [7]. The release of ROS, as well as LPS, directly leads to oxidative stress and the activation of KC, as well as the subsequent production of pro-inflammatory cytokines such as TNF-α and IL-6 [8,9,10,11,12].

As a consequence of these induced inflammatory processes, activated KC and various deleterious agents together activate HSC [13,14], which are pericytes in the perisinusoidal space. Activated HSC transform into myofibroblasts via TGF-β-dependent mechanisms [15]. Myofibroblasts also secrete pro-inflammatory cytokines [16,17,18] and lead to collagen deposition in the injured liver, which is a major step in the development of fibrosis. The activation of HSC to myofibroblasts is also accompanied by the stimulation of rho kinases, resulting in the contraction of HSC and therefore elevated intrahepatic vascular resistance [19].

Another important role in the development of fibrosis is played by LSEC, which normally comprise the first line of defense in protecting the liver from injury [20]. Inflammation by LPS or ROS results in the dysfunction of LSEC via disturbed sinusoidal microcirculation, defenestration, and pathological angiogenesis [21]. LSEC dysfunction thereby aids in the release of pro-inflammatory cytokines, hypoxia via vasoconstriction, and the activation of HSC to myofibroblasts, ending in a vicious circle of fibrosis and portal hypertension [22,23]. Different cellular pathways are involved in these pathological circumstances, such as vascular endothelial growth factor receptor (VEGF/VEGFR-), platelet derived growth factor receptor (PDGFRβ-), the mitogen-activated protein kinases (MAPK/ERK)-pathway, and the activation of transcription factors, e.g. nuclear factor-κB (NF-κB) [2,24,25].

ROS and inflammation play a critical role in the initiation and progression of chronic liver disease. However, randomized controlled clinical trials using antioxidants in alcoholic liver disease (vitamin E, *N*-acetylcysteine, coenzyme Q, and others) have failed to show efficacy in improving liver injury [26,27,28]. The cause of this discrepancy between preclinical research and clinical trials remains unclear, although additional regulatory processes may be involved.

## 2. Glyoxalase-System, MGO, AGEs and RAGE

### 2.1. The Glyoxalase-System

**Figure 1:**
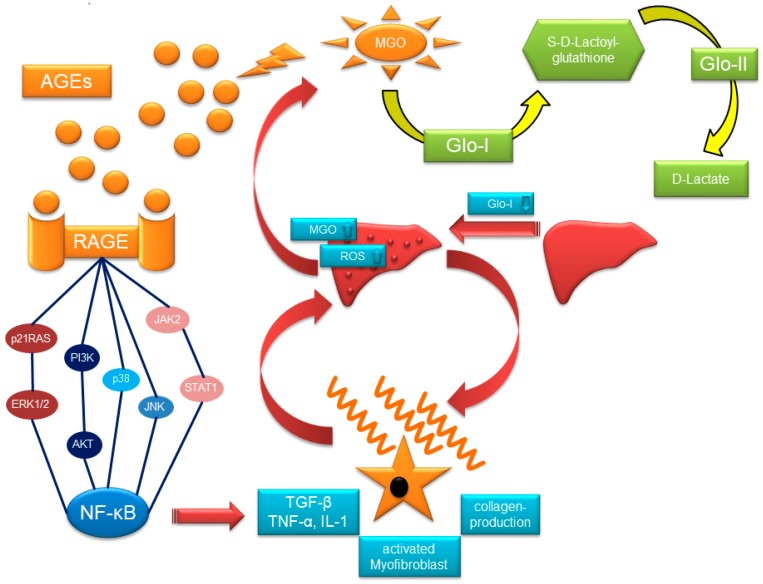
Glo-I, AGEs, and RAGE in cirrhosis. MGO reacts with proteins, nucleotides, and lipids, leading to the formation of AGEs. AGEs bind to RAGE and stimulate signal pathways (including MAPK (ERK1/2, p38, JNK), PI3-K/AKT, and JAK2/STAT1). This stimulation results in the activation of NF-κB, followed by the production of TGF-β and pro-inflammatory cytokines. These cytokines activate quiescent stellate cells, which transform to myofibroblasts and produce profibrotic factors and collagen. The collagen deposition in the liver will lead to fibrosis and finally cirrhosis. The reduction of Glo-I will perpetuate both the initiation and progression of cirrhosis through an increase of MGO and a vicious circle of disease. MGO is detoxified via Glo-I to *S*-d-Lactoylglutathione which is finally converted to d-Lactate by Glo-II. MGO: methylglyoxal. AGEs: advanced glycation endproducts. RAGE: receptor for advanced glycation endproducts. Glo-I: glyoxalase-I. Glo-II: glyoxalase II. ROS: reactive oxygen species. HSC: hepatic stellate cells. MAPK: mitogen-activated protein kinase. ERK1/2: Extracellular-signal regulated kinase. PI3-K: phosphoinositide 3-kinase. AKT: protein kinase B. JAK2: Januskinase 2. STAT1: signal transducer and activator of transcription-1. JNK: c-Jun N-terminal kinase: NF-κB: nuclear factor-κB. TGF-β: Transforming growth factor β. TNF-α: Tumor necrosis factor α. IL-1: Interleukin-1.

The glyoxalase enzymes (glyoxalase-I (Glo-I, EC 4.4.1.5) and glyoxalase-II (Glo-II, EC 3.1.2.6)) were discovered in 1913 by the research groups of Dakin-Dudley and Neuberg [29]. This enzymatic system is essential for the reduction of ROS and oxidative stress originating from dicarbonyl glycation. Glo-I catalyzes the conversion of α-oxo-aldehydes such as methylglyoxal (MGO) into the corresponding hemithioacethal *S*-d-Lactoylglutathion, using l-glutathione (GSH) as a cofactor. Further substrates of Glo-I are glyoxal (GO), hydroxypyruvaldehyde, hydroxypyruvataldehydphosphate, phenylglyoxal, 4,5-dioxovalerate, alkyl-, and arylglyoxales [30,31,32,33]. Glo-II hydrolyses the reaction of *S*-d-Lactoylglutathion to H_2_O and d-lactate, with the regeneration of GSH (Figure 1). Glo-I is therefore the rate-limiting step in this series of reactions [32,34]. The glyoxalase enzymes are found ubiquitously in all animate beings and are mainly located in the cytosol, with only a small portion also present in the mitochondria of the cell [35]. Other enzymes are, at least in part, capable of detoxifying MGO. The nicotinamide adenine dinucleotide phosphate-(NAD[P]H)-dependent aldo-keto-reductase and aldehyde dehydrogenase catalyze the NADPH-dependent reduction of MGO to hydroxyacetone, lactaldehyde, and pyruvate [36]. In addition, glyoxalase-III was discovered in *scherichia coli* and converts MGO to lactic acid in the absence of any cofactor [37]. In this regard, glyoxalase-III is a member of the DJ-1 superfamily and DJ-1 is involved in Parkinson’s disease and oxidative stress [38]. In contrast, the biochemical function of DJ-1 was not fully elucidated but recent work highlighted DJ-1 and its homologs as novel glyoxalases [39].

### 2.2. MGO and GO

MGO is the main substrate of Glo-I [40] and was found to be a reactive carbonyl compound that is formed as a by-product in glycolysis [41], ketone body metabolism, and threonine catabolism [42,43,44]. Another important substrate of Glo-I, GO, is formed by lipid peroxidation, as well as the degradation of monosaccharides, saccharide derivatives, and glycated proteins [45]. MGO and GO are highly potent glycating agents. In this regard, the glycation of proteins is a complex of multiple reactions that is also called the “Maillard reaction” involving the formation of fructosamines. Additional reactions lead to stable end-stage adducts called “advanced glycation endproducts” (AGEs). In contrast, MGO and GO can react with nucleotides, phospholipids, and proteins directly [46,47] and result in the rapid formation of AGEs. Important MGO- and GO-derived AGEs from arginine residues are hydroimidazolones (*N*_δ_-(5-hydro-5-methyl-4-imidazolon-2-yl)-ornithine (MG-H1) and *N*_δ_-(5-hydro-4-imidazolon-2-yl)-ornithine (G-H1)) [36]. Further minor MGO-related AGEs are the lysine-derived *N*^ε^-carboxyethyllysine (CEL) and lysine dimers crosslinks (MOLD), as well as argpyrimidine [48]. Additional minor GO-induced AGEs comprise the lysine-derived *N*^ε^-carboxymethyllysine (CML) and lysine dimers (GOLD) [45]. Further minor arginine-derived adducts are the MGO-mediated *N*_ω_-carboxyethylarginine and the GO-induced *N*_ω_-carboxymethylarginine [49]. Other, non-MGO-, non-GO-derived AGEs are pyrraline, 3-deoxyglucasone-lysine-dimer, and pentosidine [50]. AGEs have a yellow brown pigmentation and a characteristic fluorescence spectrum in common. Thus, AGEs can be divided into fluorescent AGEs (argpyrimidine, pentosidine, MOLD, GOLD) and non-fluorescent AGEs (CML, pyrraline, MG-H1, G-H1) [51]. Some studies have shown the feasibility of the fluorescence detection of AGEs in the serum and urine of patients [52].

As mentioned above, “dicarbonyl stress” is a result of elevated MGO and GO levels leading to AGEs. The glycation of arginine residues leads to misfolded proteins, which are directed to the proteasome for proteolysis [53]. As a consequence, mitochondrial protein dysfunction, enzyme inactivation, and cellular-mediated immune-response occur. In addition, the excessive glycation of nucleotides leads to mutagenesis and apoptosis. Excessive lipid glycation is associated with membrane lipid bilayer damage [54]. Furthermore, MGO- and GO-derived AGEs bind to their receptor RAGE and activate intracellular signaling pathways [50]. Recent work has also demonstrated that “dicarbonyl stress” can lead to oxidative stress in a very rapid way via the direct reaction with p38-MAPK and other target molecules [55]. Finally, “dicarbonyl stress” will result in elevated levels of ROS [56] and has been shown to be implicated in several ROS-mediated diseases, for instance, diabetes and vascular complications [57], cardiovascular disease [58,59], chronic renal diseases [60], and ageing and carcinogenesis [61,62].

### 2.3. RAGE and AGE-Rs

The effects of AGEs are mediated by their antagonistic receptor system. The activation of the receptor for AGEs (RAGE) leads to the generation of ROS, inflammation, angiogenesis, and proliferation [63,64]. Thereby, various signal transduction pathways are involved. The activation of RAGE stimulates the phosphorylation of the extracellular signal-regulated kinase 1/2 (ERK1/2), phosphoinositide 3-kinase (PI3-K)/protein kinase B (AKT), Januskinase 2 (JAK2), and RhoGTPases. The activation of these kinases finally results in the stimulation of NF-κB and the production of pro-inflammatory cytokines (see Figure 1) [65]. In addition, the stimulation of RAGE results in the activation of the TGF-β pathway and induces VEGF overexpression [64].

In contrast, AGE receptors (AGE-Rs) form a complex of three distinct receptors, namely AGE-R1, -R2, and -R3, which are responsible for the detoxification and clearance of AGEs [66]. AGEs and their precursors are also cleared by means of fructosamine-3-kinase, and AGE-modified proteins are lysed by the proteosomal and lysosomal proteolytic system [36]. In this regard, MGO, AGEs, and RAGE play important roles in various inflammatory processes like diabetes, aging, renal insufficiency, hypertension, and cancer [67,68,69,70,71], and Glo-I is essential for the prevention of toxic MGO-mediated effects.

### 2.4. Clinical Implication of Glo-I

Glo-I is a dimer and consists of two identical subunits with a molecular mass of 43–48 kDa [72]. Each subunit contains a zinc ion in its active center, whereas the apoenzyme remains catalytically inactive [40,73]. The active center of Glo-I is localized between both monomers, and spatial analyses reveal an octahedral arrangement of the enzyme [74]. The protein sequence of Glo-I consists of 184 amino acids with a posttranslational modification of the n-terminal met [75].

Recent studies analyzed three distinct phenotypes of Glo-I: GLO 1-1, GLO 1-2, and GLO 2-2, representing the homo- and heterozygous expression of *GLO*^1^ und *GLO*^2^ [76,77]. Demographic studies showed a higher distribution of *GLO*^1^ in Alaska and a lower *GLO*^1^ allocation in southern and eastern Europe, America, Africa, and India [78]. Furthermore, associations of distinct Glo-I phenotypes and Glo-I SNPs with diabetes [79], cardiovascular diseases [80], schizophrenia [81], autism [82,83], multiple sclerosis [84], anxiety [85], and cancer [86,87,88] were observed. These findings led to preliminary anti-tumor effects of Glo-I knockout by siRNA or enzymatic enzyme inhibition in different cancer models [89,90,91,92]. Furthermore, a Glo-I inducer formula led to improved glycemic control and vascular function in 29 obese patients [93]. Several anti-inflammatory and antitumor agents showed modulation of Glo-I expression and activity, e.g., *S*-ρ-bromobenzylglutathione or *S*-ρ-bromobenzylglutathionecyclopentyldiester [90,94], methotrexate [95], indomethacin [96], troglitazone [97], and flavanoids [98,99].

In this regard, the glyoxalase system is essential for the detoxification of MGO to prevent the formation of AGEs. Glo-I, in particular, is involved in different pathophysiological inflammatory processes.

## 3. Glo-I, AGEs, and RAGE in Chronic Liver Injury

### 3.1. Glo-I in Liver Disease

**Figure 2:**
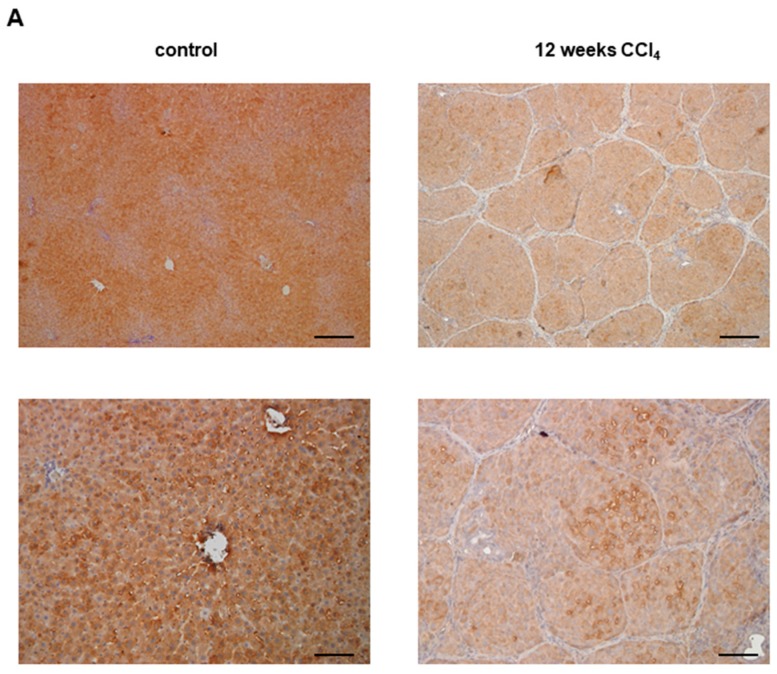

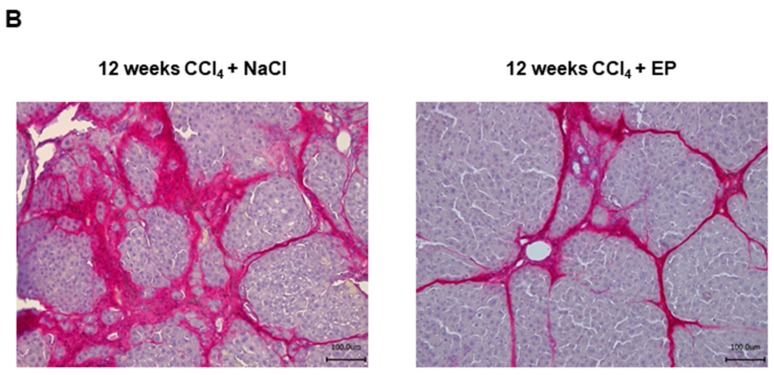
Glyoxalase-I in CCl_4_-induced cirrhosis. (**A**) Wistar rats were treated for 12 weeks, three times per week, with inhalative CCl_4_ to induce advanced cirrhosis. Explanted livers were fixed in 4% formaline, dehydrated, and embedded. Immunostaining for Glo-I with DAB compound was performed as previously described [83]. Overview sections (5× magnification, upper line) and sections at 20× magnification (lower line) showed reduced expression of Glo-I in advanced cirrhosis; (**B**) Wistar rats were treated with CCl_4_ and intraperitoneally (i.p.) EP 40 mg/kg body weight daily or saline from weeks 8–12. Explanted livers were fixed, dehydrated, and embedded. Sirius red staining was performed as previously described [83]. Sections showed reduced amount of Sirius red upon EP treatment. Scale bars: 400 µm (**A**, upper line), 100 µm (**A**, lower line, **B**).

Despite the fundamental role of Glo-I in inflammation, data about Glo-I in chronic liver diseases is not available. Therefore, our group analyzed Glo-I in a CCl_4_-model of cirrhosis [100]. Wistar rats were treated with inhalative CCl_4_ three times a week to induce early cirrhosis without ascites after eight weeks, or advanced cirrhosis with ascites after 12 weeks. Furthermore, we isolated primary liver cells from cirrhotic and noncirrhotic livers via portal vein perfusion and analyzed Glo-I. Glo-I could be detected in HEP, HSC, and LSEC, with the highest expression on protein and mRNA levels in HEP. In the CCl_4_-model of cirrhosis, Glo-I expression was reduced in early and advanced cirrhosis in both the liver as a whole and primary liver cells (Figure 2A, Glo-I DAB-staining of whole liver sections from control animals and after 12 weeks CCl_4_-treatment). Furthermore, we found a consecutive increase of MGO levels in cirrhotic livers. We were also able to show that, in addition to the reduction of Glo-I, advanced cirrhosis coincided with an elevation of RAGE (Hollenbach et al., AASLD 2015, abstract ID1519). The accumulation of MGO will subsequently lead to the increased formation of AGEs. An elevated concentration of AGEs and increased expression of RAGE will in turn result in oxidative stress with perpetuating liver inflammation [100].

### 3.2. Effects of Glo-I Modulation by Ethyl Pyruvate

To obtain further insights into the role of Glo-I in cirrhosis, we performed both in vitro and in vivo experiments with the anti-inflammatory drug ethyl pyruvate (EP), which modulates Glo-I [101]. EP is an α-oxo-carbonic acid and ester of pyruvate. Pyruvate showed anti-inflammatory effects in various animal models but was restricted by its low stability in aqueous solutions [102]. Therefore, EP constitutes a more stable compound than pyruvate and exerts anti-inflammatory and protective effects in ROS-mediated models of ischemia and reperfusion [103,104,105], hemorrhagic shock [106], septic shock [107,108], cecal ligation and perforation [109], acute renal failure [110,111], pancreatitis [112,113,114,115], thermal injury [116], brain injury [117,118,119,120,121], cardiac injury [122,123], retinal damage, and uveitis and cataract [124,125,126,127,128,129]. The effects of EP on RAGE were also analyzed in several studies, showing a reduction of RAGE expression upon EP treatment [130,131].

The molecular basis for the reduced production of TNF-α, IL-6, HMGB1, iNOS, and NO, as well as the prolonged survival in animals treated with EP, was not fully explained. Our previous work demonstrated that EP was a modulator of specific Glo-I activity, providing a new mechanism for its anti-inflammatory effects [101]. Since EP showed protective effects in acute liver failure [132,133,134,135] and in the development of fatty liver [136], we analyzed the effect of EP on activated HSC after stimulation with LPS, as it might occur in an initial stage of cirrhosis. Stimulation of HSC with LPS for 24 h led to increased levels of α-SMA and collagen-I. This stimulation could be abrogated by the modulation of Glo-I activity through EP, with underlying mechanisms involving the stimulation of Nrf2, as well as the reduction of NF-κB and ERK/pERK by EP [100]. Furthermore, we used EP in vivo: Wistar rats were treated with CCl_4_ for 12 weeks and i.p.-injected with either 40 mg/kg b.w. EP or saline. After 12 weeks, livers were stained with Sirius red, indicating collagen deposits. Rats treated with EP revealed significantly less Sirius red staining and reduced fibrosis (Figure 2B).

Based on these promising results in animal studies, a clinical phase II study analyzed EP in patients undergoing cardiopulmonary bypass surgery. In this study, 7500 mg EP i.v. was administered every 6 h to achieve a corresponding dose of 40 mg/kg b.w., as was used in many animal models. The combined endpoint was death, mechanical ventilation, acute renal failure, or the need for vasoconstrictors. No statistically significant differences were observed in the 102 patients who were studied (placebo: *n* = 53; EP: *n* = 49) with regard to clinical parameters or markers of systemic inflammation [137]. Although these first clinical results were disappointing, it should be mentioned that the chosen setting of bypass surgery might be inadequate to test the shown effects of EP from animal models.

In summary, targeting Glo-I with EP in cirrhosis demonstrated a promising therapeutic option and offers an innovative target in liver disease induced by inflammation (Figure 1).

### 3.3. AGEs in Liver Disease

The demonstrated role of Glo-I, AGEs, and RAGE in inflammatory processes would also suggest an involvement of AGEs in inflammatory liver disease. Indeed, several research groups analyzed AGEs in liver fibrosis, cirrhosis, and non-alcoholic steatohepatitis (NASH). Ahmed et al. examined protein glycation, oxidation, and nitrosation marker residues, as well as free adducts in portal, hepatic, and peripheral venous blood plasma of cirrhotic patients. They found an significant increase of MGO-derived AGEs (CEL, MG-H1), as well as GO-induced AGEs (CML, G-H1), in cirrhotic subjects compared to controls [138]. Another work showed significantly elevated concentrations of fluorescent AGEs and CML in plasma samples of patients with liver injury. Interestingly, CML levels correlated with the severity of liver disease [139]. In addition, Yagmur et al. found increased concentrations of CML in fibrosis and cirrhosis [140], and AGEs measured by fluorescence spectroscopy were also significantly elevated in cirrhosis compared to controls [141].

Recent works have tried to analyze the molecular mechanism for the observed elevated levels of AGEs in fibrosis and cirrhosis. In vitro treatment of HSC with AGEs resulted in the enhanced production of oxidative stress, providing evidence of AGEs’ involvement in liver inflammation [142]. Conversely, oxidative stress was found to elevate levels of CML in rats [143]. Thereby, the incubation of HSC with AGEs led to the elevation of α-SMA, TGF-β, and collagen-I [144]. The treatment of rat hepatocyte cultures with AGEs resulted in reduced cell viability, and the administration of ethanol to Wistar rats led to elevated levels of AGEs in rat livers [145]. In a translational study, a positive correlation of CML-AGEs with liver stiffness as an indicator for fibrosis in patients with chronic hepatitis C was found (*r* = 0.5731, *p* < 0.001). In vitro data of this work revealed enhanced cell proliferation of HSC treated with BSA-AGEs (CML) and an increased production of α-SMA. Furthermore, AGEs were found to induce autophagy, which subsequently contributed to fibrosis in patients with chronic hepatitis C [146]. These results were supported by the finding that the inhibition of CML resulted in the attenuation of CML-induced levels of α-SMA and ROS in HSC [147].

In contrast, another study showed that intraperitoneally administered AGE-rat serum albumin (CML) resulted in increased levels of α-SMA without having an influence on fibrosis. However, the additional administration of AGE-rat serum albumin to rats that underwent bile-duct ligation for the induction of fibrosis showed increased hydroxyproline, Sirius red content, and α-SMA, indicating elevated fibrosis [148].

AGEs have also been implicated in fibrosis in models of NASH. Hepatic steatosis showed the accumulation of CML. Also, CML was associated with a higher grade of hepatic inflammation and an elevated expression of inflammatory markers (PAI-1, IL-8, and CRP) [149]. AGEs have also been shown to be involved in the pathogenesis of insulin resistance and diabetes, which are risk factors for the development of NAFLD [150]. Rats fed with a diet rich in AGEs showed elevated oxidative stress and hepatic inflammation leading to NASH [151]. Additionally, rats that were subjected to a diet rich in AGEs showed increased hepatic AGEs levels, increased liver injury, inflammation, and liver fibrosis via oxidative stress in activated HSC [152]. Recently, the molecular basis for the involvement of AGEs in NASH was discovered. AGEs induce NOX2, leading to a downregulation of Sirt1/Timp3 and finally resulting in the activation of TNF-α converting enzyme and inflammation [153].

Keeping the growing evidence of AGEs in fibrosis and chronic liver disease in mind, several studies analyzed the effect of AGEs-reduction on inflammation and fibrosis in NASH. Tang et al. found that the anti-inflammatory drug curcumin eliminated the effects of AGEs in HSC by interrupting leptin signaling and activating the transcription factor Nrf2, which led to the elevation of cellular glutathione and the attenuation of oxidative stress [154]. Also, curcumin showed reduced AGEs-induced activation and proliferation of HSC, as well as induced gene expression of AGE-clearing receptor AGE-R1 [155].

In another study, the LDL-lowering drug atorvastatin decreased levels of AGEs in patients with NASH and dyslipidemia, leading to improved steatosis and nonalcoholic fatty liver disease activity scores [156]. Miura et al. was able to show that a combination therapy of telmisartan and nateglinide reduced levels of AGEs in rats and ameliorated insulin resistance [157]. Another approach evaluated the effects of aqueous extracts from Solanum nigrum (AESN). AESN reduced the AGE-induced expression of collagen-II, MMP-2, and α-SMA in HSC. AESN also improved insulin resistance, as well as hyperinsulinemia and downregulated lipogenesis, finally preventing fibrosis [158].

Thus, there is growing evidence for the involvement of MGO-related AGEs in chronic liver disease. Nevertheless, mainly CML was investigated in the aforementioned studies. Therefore, it should be considered that CML-AGEs are rarely produced via the reaction of MGO and are more likely to be formed in lipoxidation and glycation via GO [159].

### 3.4. RAGE in Liver Disease

RAGE is a pattern recognition multi-ligand cell surface receptor that belongs to the immunoglobulin superfamily with a molecular mass of 47 to 55 kDa. RAGE expression is usually low but can be elevated under inflammatory conditions such as diabetes, cardiovascular diseases, or cancer [160]. RAGE has been shown to be activated by MGO- and non-MGO-derived AGEs, and the activation of RAGE leads to intracellular signaling cascades resulting in inflammation, proliferation, and angiogenesis mediated by NF-κB [161].

Several studies analyzed the impact of RAGE in chronic liver disease: Goodwin et al. generated AGE-rat serum albumin (mainly CML) and illustrated that treatment with AGE-rat serum albumin resulted in increased oxidative stress. Interestingly, levels of RAGE, α-SMA, hydroxyproline, and Sirius red were stimulated in a fibrosis model of bile-duct ligation (BDL) if the animals received additional AGE-rat serum albumin [148]. Another study confirmed the expression of RAGE predominantly in HSC. RAGE was stimulated in HSC during transformation to myofibroblasts. In addition, RAGE was colocalized with α-SMA and induced by TGF-β. Since a high expression of RAGE was found in filopodial membranes of myofibroblasts, this suggests that RAGE plays a role in the spreading and migration of activated HSC in fibrogenesis [162]. Also, an elevated expression of RAGE was confirmed in activated HSC and LSEC in a fibrosis model of bile-duct-ligation. RAGE-expression was significantly increased through AGE-serum albumin and TNF-α, but it did not affect HSC proliferation, apoptosis, or fibrosis signal transduction [163]. Serban et al. further analyzed the regulation and crosstalk of RAGE in fibrosis. They concluded that AGEs-induced RAGE upregulation resulted in the induction of TGF-β, TNF-α, and IL-8. Furthermore, it was propagated that there is an inhibitory crosstalk between TGF-β and RAGE, since RAGE also stimulated the anti-inflammatory cytokines IL-2 and IL-4 [164].

To further analyze the role of RAGE in fibrosis, recent studies investigated the effects of RAGE inhibition. Firstly, the anti-inflammatory drug curcumin (also reducing AGEs, see above) inhibited the AGEs-induced gene expression of RAGE via the elevation of PPAR-γ [165]. In addition, RAGE expression was diminished by means of RAGE siRNA in primary rat HSC, resulting in the downregulation of IL-6, TNF-α, and TGF-β [166]. The authors of this study conducted a subsequent in vivo approach, analyzing the effect of RAGE siRNA in an olive-oil model of fibrosis. RAGE siRNA was injected twice weekly in the tail vein of Sprague-Dawley rats. After six weeks, reduced expressions of RAGE, TNF-α, IL-6, extracellular matrix, hyaluronic acid, and procollagen III were found. Also, the activation of HSC and NF-κB was reduced in siRNA-treated animals, attenuating the initiation and progress of fibrosis [167]. Additional studies revealed protective effects of anti-RAGE antibodies in BDL-induced acute liver injury [168,169].

In addition to its implication in both BDL and pharmacological models of fibrosis, RAGE is also involved in the development of non-alcoholic fatty liver disease (NAFLD). A methionine cholin deficient (MCD) diet caused steatosis in mice and significantly increased RAGE, pro-inflammatory cytokines, and fibrosis [152]. Recently, fatty acids stimulated CML accumulation and subsequently elicited RAGE induction [149]. Another research group found an upregulation of RAGE in the liver of aged mice with consecutive elevated oxidative stress shown by the analysis of malondialdehyde. Blocking of RAGE by an anti-RAGE-antibody revealed the prolonged survival of animals in this work [170].

These findings suggest that the activation of RAGE is a major driver of fibrosis, and RAGE inhibition could prevent the initiation and progress of extracellular matrix deposition, as shown in Figure 1.

## 4. Glo-I/AGE/RAGE in HCC

Hepatocellular carcinoma (HCC) constitutes the sixth most common cancer worldwide and the third most common cause of cancer-related mortality [171]. About 90% of HCC stem from the development of cirrhosis, thereby making cirrhosis the most important risk factor for the development of HCC [172]. During tumorigenesis, the dysregulation of cell proliferation, invasion, metastasis, and angiogenesis occur. These alterations were indicated, amongst others, by an elevated expression of transcription factors IGF and FGF [173], Snail [174], PDGF [175], and VEGF [176].

Recent work analyzed the role of Glo-I in hepatocellular carcinoma (HCC). Glo-I mRNA was upregulated in tissue samples of HCC patients. Therefore, Glo-I knockdown by means of siRNA resulted in the reduced proliferation of Hep3B, SK-HEP-1, and SMMC-7721 HCC cell lines and was accompanied by elevated levels of MGO [177]. Another study revealed genetic amplification and upregulation of Glo-I in HCCs. Knockdown of Glo-I through sh-RNA led to the inhibition of tumor growth and the induction of apoptosis in primarily cultured HCC [178]. Nevertheless, the distinct role of Glo-I in HCC remains preliminary and needs to be confirmed in additional studies.

In contrast, AGEs and RAGE have been intensively studied in HCC: serum levels of AGEs were elevated in patients with HCC without hepatitis B or C infection. AGEs were significantly higher in HCC patients compared with NASH and control subjects (9.1 ± 2.7, 5.2 ± 1.7, 3.5 ± 1.2 U/mL. *p* < 0.05) [179]. Furthermore, levels of the soluble form of RAGE (sRAGE) were shown to predict tumor progression in HCC patients undergoing transarterial chemoemobilisation (TACE) in a first proof-of-concept study [180]. Another translational study confirmed the overexpression of RAGE and sRAGE in HCC in a small cohort of 10 patients and showed reduced cell proliferation and DNA synthesis upon RAGE knockdown via siRNA. Also, stimulation of RAGE with the ligand HMGB1 stimulated cell proliferation and the activation of NF-κB in Huh7 cells [181]. Several additional studies showed the importance of RAGE for proliferation [182], angiogenesis [183], and invasion [184] of HCC and confirmed reduced tumor growth by means of RAGE inhibition [185,186]. In contrast, in a case-control-cohort of hepatitis-related HCC, levels of sRAGE and CML-AGEs were inversely associated with HCC development [187]. This study showed several limitations, mainly due to the fact that only men and smokers were included. Nevertheless, further analysis, particularly in larger populations, is necessary.

In conclusion, Glo-I is responsible for the detoxification of MGO and reveals an essential role for the prevention of the MGO-induced formation of AGEs and binding to RAGE. Recent work has highlighted the role of Glo-I in cirrhosis, and the importance of RAGE and AGEs in fibrosis and HCC has been proven. Therefore, the Glo-I/AGE/RAGE system indicates an innovative and promising target in treatment for cirrhosis and chronic liver disease.

## Abbreviations

AGEs	Advanced glycation endproducts
EP	Ethyl pyruvate
Glo-I	Glyoxalase-I
Glo-II	Glyoxalase-II
G-H1	*N*_δ_-(5-hydro-4-imidazolon-2-yl)-ornithine
GO	Glyoxal
GOLD	Glyoxal lysine dimer
GSH	l-glutathione
HCC	Hepatocellular carcinoma
HEP	Hepatocytes
HSC	Hepatic stellate cells
KC	Kupffer cells
LSEC	Liver sinusoidal endothelial cells
MG-H1	*N*_δ_-(5-hydro-5-methyl-4-imidazolon-2-yl)-ornithine
MGO	Methylglyoxal
MOLD	Methylglyoxal lysine dimer
miRNA	microRNA
NAFLD/NASH	Non-alcoholic fatty liver disease/steatohepatitis
RAGE	Receptor for advanced glycation endproducts
sRAGE	Soluble form of RAGE
ROS	Reactive oxygen species
